# EFFECT OF AWARENESS OF HEARING LOSS AMONG CARE RECIPIENTS ON ATTRIBUTES OF CARE PROVIDED: A NATIONAL STUDY

**DOI:** 10.1093/geroni/igae098.2529

**Published:** 2024-12-31

**Authors:** Danielle Powell, Wuyang Zhang, Nicholas Reed

**Affiliations:** University of Maryland, College Park, Maryland, United States; Johns Hopkins University, Baltimore, Maryland, United States; Johns Hopkins University, Baltimore, Maryland, United States

## Abstract

Hearing loss (HL) is prevalent among older adults and may result in caregiving challenges due to communication barriers. This study aims to investigate whether care recipients’ awareness of HL is associated with attributes of care. Using data from Round 12 (2022) of the linked National Health and Aging Trends Study (NHATS) and National Study on Caregiving (NSOC), a sample of 1,133 (Mean age: 62±15 years; Female: 68%) caregivers assisting 713 older adults (Mean age: 83±8 years; Female: 63%; White: 63%) with audiometric HL was derived. From care recipients’ self-reported hearing difficulty and hearing aid use, HL awareness was defined as (1) unrecognized HL (no hearing difficulty or hearing aid use reported), (2) recognized HL (only hearing difficulty reported), and (3) managed HL (hearing aid use reported). Caregiving outcomes, including ADL/IADL-related care needs and past month hours of caregiving, were measured through self-report. Of all caregivers, 628 (55%) assisted older adults with unrecognized HL, while 162 (15%) and 343 (30%) assisted older adults with recognized and managed HL, respectively. Relative to unrecognized or managed HL, care recipients with recognized HL reported greater needs on ADL/IADL-related activities (≥3 ADL needs: 65.4% vs. 47.8%/45.0%; IADL needs: 55.1% vs 48.3%/40.0%). Results from models adjusted for multiple caregiver-recipient characteristics suggest time spent on care activities by caregivers of those with recognized HL and managed HL were 0.66 (95%CI:0.43-1.03) and 0.84 (95%CI:0.56-1.27) times lower than those with unrecognized HL. Supporting HL awareness and management as aligned with care needs may contribute to effective provision of care.

